# Identifying potential factors associated with PCR testing for COVID-19 among Australian young people: cross-sectional findings from a longitudinal study

**DOI:** 10.1186/s12889-022-14892-1

**Published:** 2022-12-23

**Authors:** Md Irteja Islam, Verity Chadwick, Alexandra Martiniuk

**Affiliations:** 1 Sydney School of Public Health, Faculty of Medicine and Health, The University of Sydney, Edward Ford Building, A27 Fisher Road, Camperdown, Sydney, NSW 2006 Australia; 2 Centre for Health Research and Faculty of Health, Engineering and Sciences, The University of Southern Queensland, West Street, Darling Heights, Toowoomba, QLD 4350 Australia; 3 Royal North Shore Hospital, Reserve Rd, St Leonard’s, Sydney, NSW 2065 Australia; 4 Office of the Chief Scientist, The George Institute for Global Health, Level 5/1 King Street, Newtown, Sydney, NSW 2042 Australia; 5 Dalla Lana School of Public Health, The University of Toronto, 155 College St Room 500, Toronto, ON M5T 3M7 Canada

**Keywords:** COVID-19, Coronavirus, COVID testing, Australia, Adolescents, Young adults

## Abstract

**Background:**

Testing has played a crucial role in reducing the spread of COVID-19. Though COVID-19 symptoms tend to be less severe in adolescents and young adults, their highly social lifestyles can lead to increased transmission of the virus. In this study, we aimed to provide population-based estimates of polymerase chain reaction testing (PCR) for the COVID-19 pandemic and identify factors associated with PCR testing in Australian youth using the latest survey data from the Longitudinal Study of Australian Children (LSAC).

**Methods:**

We used the latest wave (9C1) of the LSAC, collected from 16 to 21-year-old Australians via an online survey between October and December 2020. In total, 2291 youths responded to the questions about COVID-19 testing including factors related to the coronavirus restriction period (CRP) in Australia. Both bivariate and multivariate logistic regression analyses were performed to identify variables (sociodemographic factors and factors related to CRP) associated with COVID-19 testing.

**Results:**

During the study period, 26% (*n* = 587) of Australian youth aged between 16 and 21 years were tested for COVID-19. The strongest predictor of COVID-19 testing was living in major cities (aOR 1.82, 95% CI:1.34–2.45; *p* < 0.01). Increased age (aOR 1.97, 1.00–3.89; *p* < 0.05) and having a pre-existing medical condition (aOR 1.27, 1.02–1.59; p < 0.05) were also significantly associated with a higher likelihood of COVID-19 testing.

**Conclusion:**

Age, remoteness and having a pre-existing medical illness were associated with PCR COVID-19 testing among Australian youth aged between 16 and 21 years in the first year of the COVID-19 pandemic. More research is warranted to identify factors associated with other COVID-19 testing methods and address the specific barriers that may limit COVID-19 testing in this age group.

**Supplementary Information:**

The online version contains supplementary material available at 10.1186/s12889-022-14892-1.

## Background

Testing, tracking, and isolation/quarantine (TTIQ) play crucial roles in epidemic control, which requires fast and comprehensive testing and tracing, and sufficient compliance with isolation/quarantine [1]. Australia relied only on Polymerase Chain Reaction (PCR) tests until November 2021, with PCR tests free of charge if individuals self-report COVID-19 symptoms or self-reported COVID-19 close contacts. In Australia, regular testing was mandatory between 2020 and 2021 for some workplaces e.g., warehouses/factories in areas with disproportionately high caseloads, and before travel [2, 3].

Some studies of factors associated with COVID-19 testing have been published in other populations. In the USA, factors associated with testing included: free testing, concern about health, and having a high number of social contacts [4, 5]. Interestingly, in a study using hypothetical scenarios, individuals said cost and ability to support oneself if needing to isolate were of no concern and did not influence their likelihood to get tested [4]. However, in practice, American Latino and Black respondents, and those experiencing financial strain were disproportionately more likely to indicate that resource factors would influence their decision to get tested [5]. This effect remained after controlling for not having health insurance, experiencing material hardship, and general financial constraints [5]. Such findings are consistent with other research [6, 7] indicating that the basic survival needs of individuals and families often outweigh other considerations in healthcare decision-making, including prosocial motives like avoiding transmission of infection to others.

It is well established that all people, but more so children and young people, can be asymptomatic carriers of SARS-CoV-2 [8]. Although COVID-19 symptoms and outcomes tend to be less severe in children and adolescents, a key concern is that young people may be important community reservoirs for the transmission of the virus to household members, grandparents, and other community members. Further, adolescents and young adults tend to have a higher number of daily in-person encounters, [4] and often live in crowded houses [9] and work multiple jobs [10]. These factors might be playing a crucial role in increasing SARS-COV-2 transmission in the community [11–13].

Throughout the whole pandemic period to date, just under half of Australians reported they would get a COVID-19 test if experiencing mild respiratory symptoms [14, 15]. Statistics from the Australian state of New South Wales (NSW) suggest that approximately one-quarter of daily COVID-19 tests have been for individuals aged 10–29 years of age [16]. Although testing is crucial for all age groups, testing to reduce transmission is arguably more vital in the highly social and mobile adolescent and young adult age group [4]. However, there is a shortage of evidence regarding factors associated with adolescents and young adults seeking COVID-19 testing across the world, including Australia, as most studies considered adult populations and/or healthcare providers as their sample [17–19]. A previous study in Australia conducted interviews with 14 health professionals regarding barriers to COVID-19 testing for all ages [18]. To the best of our knowledge, no study has yet provided population-based estimates regarding COVID-19 testing in adolescents and young adults from a nationally representative sample.

This study, therefore, aimed to provide population-based estimates of PCR COVID-19 testing (regardless of exposure to the SARS-COV-2 virus or COVID-19 symptoms at the time of testing) in Australian adolescents and young adults aged 16–21 years. This study also aimed to identify factors associated with the higher likelihood of self-reported PCR COVID-19 testing, using the latest survey data (i.e., Wave 9C1) from the Longitudinal Study of Australian Children (LSAC). Study data in the latest wave were collected in 2020 (including the first coronavirus lockdown period in Australia between March and May 2020). Further, because of the distribution of COVID-19 infections in Australia in the first year of the pandemic as well as the availability of PCR testing sites we hypothesized that living in a major city would be a significant predictor of PCR COVID-19 testing in young adults during the first year of the COVID-19 pandemic.

## Methods

This study is reported following the the Strengthening the Reporting of Observational Studies in Epidemiology (STROBE) guidelines (Supplementary file 1) [20].

### Study design and sample selection

We used data from Growing Up in Australia: The Longitudinal Study of Australian Children (LSAC), a population-based cross-sequential cohort study carried out by the Australian Government Department of Social Services (DSS) in partnership with the Australian Institute of Family Studies (AIFS) and the Australian Bureau of Statistics (ABS). The LSAC sampled participants from the Medicare enrolment database, Australia’s universal health insurance scheme. Following a multi-stage cluster sampling technique (first stratification, then clustering and finally, weighting) on a complex probability sample to provide credible population estimates [21], the LSAC collected data biennially from the participating families for two cohorts in 2004. The younger B-cohort (aged 0–1 year at baseline, *n* = 5107) and the older K-cohort (aged 4–5 years at baseline, *n* = 4983). In total, 10,090 children were recruited during the baseline survey (termed Wave 1 by the LSAC) in 2004, and in the following waves, data were gathered from the same participants as they aged from 2004 to 2020. More details on the LSAC methodology, including sampling procedures and data collection techniques, can be found elsewhere [21, 22]. In the LSAC Wave 9C1, 2956 participants (B-cohort = 1595 and K-cohort = 1361) responded during the COVID-19 pandemic between October and December 2020.

Since, we were interested in COVID-19 testing, we only used LSAC 9C1 wave in this study because older are more socially active and have a more independent life compared to younger ages, as well as because the latest LSAC cohort survey only collected data from 16 to 21-year-olds and their parents. Respondents who reported on the outcome variable (COVID-19 testing) and explanatory variables were only included in the analyses, while the 665 non-response categories (B-cohort = 221 and K-cohort = 444) were omitted.

### Measures

The LSAC Wave 9C1 online survey questionnaires included questions related to the COVID-19 pandemic (including the coronavirus restriction period between March–May 2020) and sociodemographic characteristics among the parents and/or youths.

### Outcome variable

The question related to testing for COVID-19 included whether the youth had been tested for severe acute respiratory syndrome coronavirus 2 (SARS-CoV-2) with response categories ‘Yes’ (coded as 1) and ‘No’ (coded as 0). Note that PCR tests were only used to diagnose COVID-19 in Australia until November 2021. There were no “home tests”/ rapid antigen (lateral flow) tests available in Australia during the study period, and PCR tests were free of cost. The LSAC did not collect data on COVID-19 exposure and/or COVID-19 symptoms at the time of PCR testing.

### Explanatory variables

Based on the previous literature, [23, 24] the following socio-demographic variables were considered as variables of interest in this study: age (16–17 years, 20–21 years), sex (Male, Female), remoteness (Major cities, Rural/remote areas), education (Technical/others, Secondary, Diploma and above), employment (Employed, Unemployed), number of household members (Less than two people, 3-4 people, Five or more people), living with parents (Yes, No), currently in a relationship (Yes, No), and the SEIFA (Socio-Economic Indexes for Areas) Index of Relative Socio-Economic Advantage and Disadvantage (IRSAD) quintiles. For all areas across Australia, IRSAD quintile 1 includes the lowest 20% for the most disadvantaged areas, and quintile 5 contains the highest 20% for the most advantaged areas [25]. Regarding family cohesion, in general, the youth were directly asked to rate the ability of a family or household members to get along with each other where the responses were recorded on a 5-point scale (from Excellent to Poor) [26]. However, for analytical purposes, we created a dichotomized variable – ‘cohesion among household members’ from the responses. Youth who responded ‘excellent’, ‘very good’ or ‘good’ family cohesion were classified as ‘yes’ (coded as 0), while those who answered, ‘fair’ or ‘poor’ were classified as ‘no’ (coded as 1). ‘Any medical condition’ was also included as one of the variables of interest. When a youth reported a medical condition that lasted for the past 6 months or more or reported requiring help or supervision for mobility, responses were coded as 1 for ‘yes’, otherwise coded as 0 for ‘no’ medical condition. Medical conditions included sight problems not corrected by glasses or contact lenses, difficulty in learning and/or understanding, restricted use of limbs, less physical activity or disfigurement or deformity, and any mental illness.

Since the respondents of LSAC Wave 9C1 experienced the COVID-19 pandemic in 2020, the study included COVID-19 and coronavirus restriction period (CRP) variables between March–May 2020 in Australia [22]. Our study thus included the following additional variables: employment status during CRP (Employed, Unemployed), changed household composition during CRP (Yes, No), and received coronavirus supplement^1^ during CRP (Yes, No) to assess whether these variables were associated with COVID-19 testing among the respondents. The following question was directly asked for youth in W9C1 about the changes in household composition since the previous LSAC W8 in 2018 as the data were rolled forward: ‘Whether the composition of household changed (any person added or moved out) during the COVID-19 restriction period, except youth and their parents, since the previous wave?’ Note that the regular detailed household data could not be gathered in W9C1 since the questions were meant to be asked in a face-to-face interview (which was not possible during CRP) and could not be fully converted into online inquiries [22].

### Statistical analysis

Initially, descriptive statistics in terms of frequencies (n) and percentages (%) were computed for the total sample population. Then, we conducted bivariate analyses using Pearson’s chi-square test [27] [28] to examine the predictor variables and their distributions over the outcome variable (i.e., COVID-19 testing). Next, we employed logistic regression analysis to identify predictors of COVID-19 testing. Factors associated with COVID-testing in the bivariate analysis with two-sided *p*-value less than 0.05 were considered statistically significant and adjusted in the logistic model. The results of the regression analyses were presented as adjusted odds ratios (aOR) with corresponding 95% confidence intervals (CI). As recommended by the LSAC data user guide for Wave 9C1, [22] we used the ‘SVY’ command [29] of Stata to take into account the LSAC’s complex survey design that included stratification, clustering and weighting, and to deal with potential non-response bias and to avoid overestimation of statistical significance.

The assumptions of the logistic regression model were assessed using several statistical diagnostic tests. For instance, McKelvey & Zavoina’s R^2^ test [30] and Goodness-of-fit statistics [31] [32] were used to determine model fitness. In addition, the Variance inflation factor (VIF) test [33] was used to assess multicollinearity among predictor variables. All statistical analyses were conducted with Stata/SE 14.1.

### Ethics

The LSAC has been approved by the Human Research Ethics Committee of the Australian Institute of Family Studies (AIFS) (Application number 20–09), and written informed consent was obtained for all study participants. In addition, the authorship team obtained permission from Australian Data Archive Dataverse to use LSAC data for research and publications (Reference No. 263493).

## Results

**Fig. 1:**
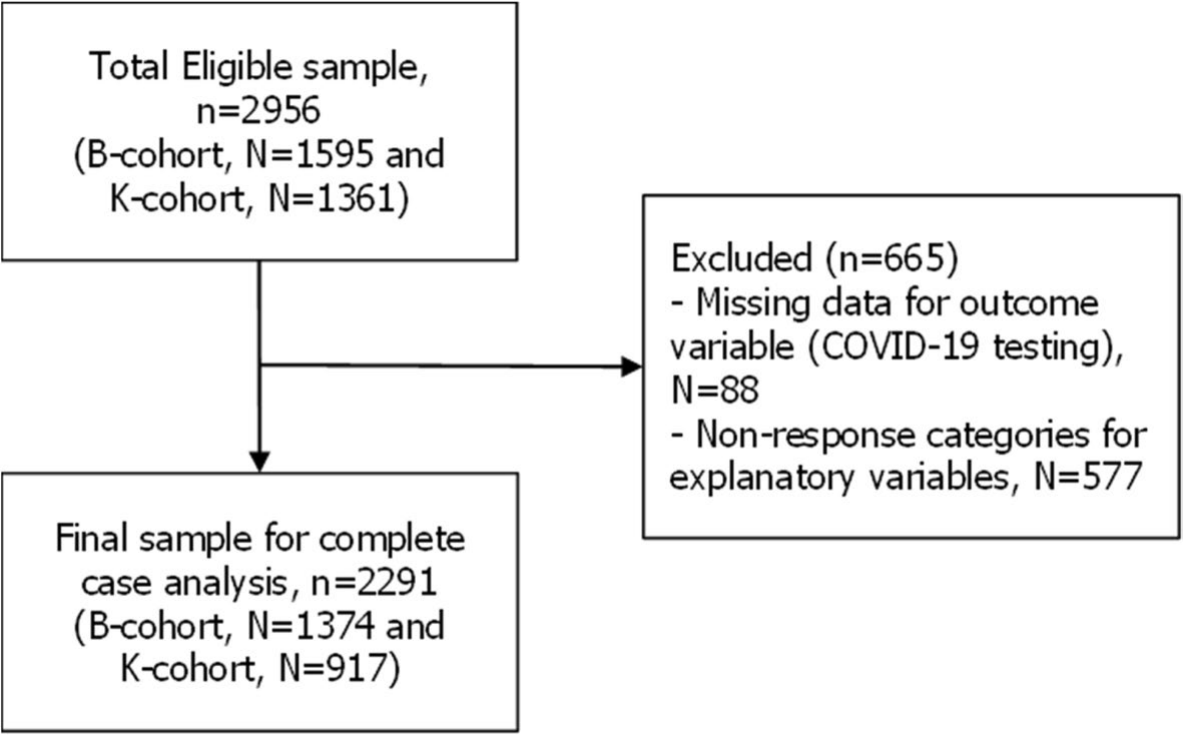
Flow diagram for the selection of analytical sample

Our current study included 2291 Australian youth aged 16–21 years (B-cohort = 1374 and K-cohort = 917) at the time of the LSAC Wave 9C1 in 2020. Fig. 1 shows the flow diagram for the selection of the final analytical sample.

Descriptive statistics of the study population are demonstrated in Table 1. Of the total sample, 60% (*n* = 1374) were aged between 16 and 17 years, more than 56% (*n* = 1301) were female, 97% (*n* = 2232) were born in Australia, 73% (*n* = 1667) were from major cities, 57% (*n* = 1300) had secondary level educational qualifications, and 57% (*n* = 1320) had part-time employment. About 75% (*n* = 172) of youth had three or more household members, 84% (*n* = 1919) reported cohesion among the household members, 88% (*n* = 2015) of youth were living with parents and 30% (*n* = 698) were in a relationship. Nearly 70% of respondents were from the disadvantaged group [Quintile 1 (*n* = 556, 24.3%), Quintile 2 (*n* = 465, 20.3%), and Quintile 3 (*n* = 548, 23.9%) combined]. Around 24% (*n* = 534) of the analytical sample reported having a medical condition that lasted for more than 6 months prior to the survey and needed help or supervision for that condition. During the coronavirus restriction period (CRP) between March–May 2020, more than 50% (*n* = 1215) of youth were unemployed, most of the respondents (*n* = 1930, 84%) did not experience changed household composition, and 82% (*n* = 1881) of youth did not receive the coronavirus financial supplement.

**Table 1:** Sample characteristics (*n*=2291)

Variables	*n* (%)
Age (in years) [Mean=18.24, SD=2.00]	
16-17	1374 (60.0)
20-21	917 (40.0)
Sex	
Male	990 (43.2)
Female	1301 (56.8)
Remoteness	
Rural/Remote	624 (27.2)
Major cities	1667 (72.8)
Education	
Technical/Others	175 (7.7)
Secondary	1300 (56.7)
Diploma and above	816 (35.6)
Employment status^1^	
Unemployed	819 (35.8)
Employed (part-time/full-time)	1472 (64.2)
Number of household members	
Less than two	571 (24.9)
Three-four	1404 (61.3)
More than five	316 (13.8)
Living with parents	
No	276 (12.1)
Yes	2015 (87.9)
Cohesion among household members^2^	
No	372 (16.2)
Yes	1919 (83.8)
Currently in a relationship	
No	1593 (69.5)
Yes	698 (30.5)
IRSAD Quintiles^3^	
Q1 (0-20%) - Most disadvantaged	556 (24.3)
Q2 (20-40%)	465 (20.3)
Q3 (40-60%)	548 (23.9)
Q4 (60-80%)	330 (14.4)
Q5 (80-100%) - Most advantaged	392 (17.1)
Any medical condition^4^	
No	1757 (76.7)
Yes	534 (23.3)
Employment status during CRP^5^	
Unemployed	1215 (53.0)
Employed (part-time/full-time)	1076 (47.0)
Changed household composition^6^ during CRP	
No	1930 (84.2)
Yes	361 (15.8)
Received coronavirus supplement^7^ during CRP	
No	1881 (82.1)
Yes	410 (17.9)

^1^ An individual aged 15 years and above are entitled to work (full-time/part-time) in Australia according to the Australian Labour Force

^2^ Cohesion - the ability of a family or household members to get along with each other

^3^ IRSAD - Index of Relative Socioeconomic Advantaged and Disadvantaged, is a general measure of both relative socioeconomic advantage and disadvantage at the area level. It uses a range of different Census variables including income, education, employment, occupation and housing characteristics

^4^ Whether the youth have any conditions that lasted or are likely to last for 6-months or more (e.g., sight problems not corrected by glasses or contact lenses, difficulty learning or understanding things, limited use of limbs, any condition that restricts physical activity or disfigurement or deformity, and any mental illness for which help or supervision is required).

^5^ CRP - Coronavirus Restriction Period, between March and May 2020

^6^ Changed household composition - Whether the composition of household changed (any person added or moved out) during COVID-19 restriction except youth and their parents since the previous wave

^7^ Coronavirus supplement - Additional income support during the COVID-19 pandemic from the Australian Government for people aged 15 and over, which included Youth Allowance for students and apprentices, and Youth Allowance for Jobseeker and/or JobKeeper

**Fig. 2:**
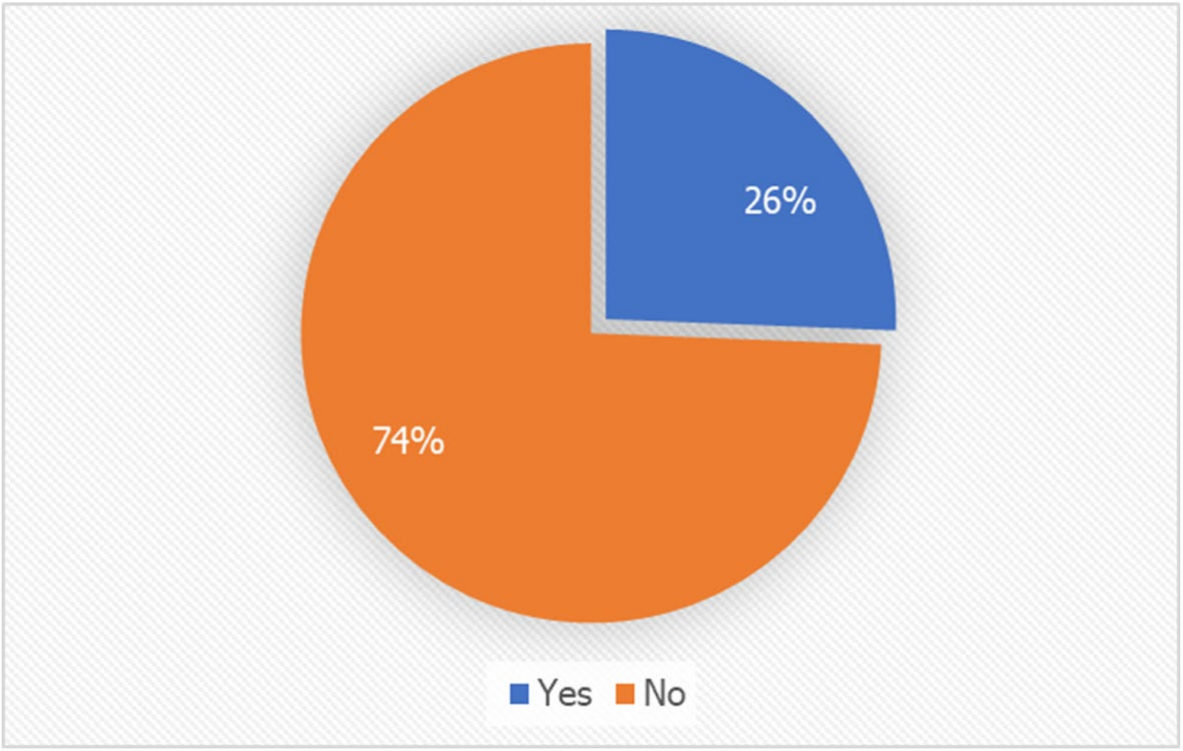
Overall % of COVID-19 testing

Fig. 2 displays the percentages of youth who were tested for COVID-19 (regardless of exposure to the virus) in the whole sample population. Overall, a quarter of youth (*n* = 587, 26%) were tested for COVID-19.

Bivariate relationships between explanatory variables and COVID-19 testing among youth are reported in Table 2. Among those who were tested 54% (*n* = 318) were aged 16–17 years compared to the age group 20–21 years (*p* = 0.001). Youth who were living in major cities compared to those living in rural/remote areas (*n* = 462, 78.7% vs *n* = 125, 21.3%; *p* < 0.001), and those who completed at least secondary education compared to their counterparts (*n* = 350, 59.6% vs *n* = 237, 40.4%; *p* = 0.01) were also found to have a significantly higher probability of having had a COVID-19 test. Table 2 also shows that the majority (*n* = 499, 85%) of youth who were tested for COVID-19 were living with their parents and only 15% (*n* = 88) were not living with their parents (*p* = 0.011). Having a pre-existing medical condition (*n* = 152, 25.9%) was significantly associated with COVID-testing (*p* = 0.036). The bivariate analysis also found that employment status during the coronavirus restriction period (CRP) (aka “lockdown”) (*n* = 255, 43.4%; *p* = 0.049), changed household composition during the CRP (*n* = 121, 20.6%; p = < 0.001) and receiving the financial coronavirus supplement during the CRP (*n* = 124, 21.1%; *p* = 0.018) were also found to be significantly associated with a higher likelihood of COVID-19 testing in Australian youths.

**Table 2:** Bivariate associations between factors and COVID-19 testing

Variables	COVID-19 tested		
	No, *n* (%)	Yes, *n* (%)	*p*-value
Age (in years)			0.001**
16-17	1056 (62.0)	318 (54.2)	
20-21	648 (38.0)	269 (45.8)	
Sex			0.459
Male	744 (43.7)	246 (41.9)	
Female	960 (56.3)	341 (58.1)	
Remoteness			<0.001***
Rural/Remote	499 (29.3)	125 (21.3)	
Major cities	1205 (70.7)	462 (78.7)	
Education			0.010*
Technical/Others	127 (7.4)	48 (8.2)	
Secondary	998 (58.6)	302 (51.4)	
Diploma and above	579 (34.0)	237 (40.4)	
Employment status^1^			0.200
Unemployed	622 (36.5)	197 (33.6)	
Employed (part-time/full-time)	1082 (63.5)	390 (66.4)	
Number of household members			0.146
Less than two	407 (23.9)	164 (27.9)	
Three-four	1058 (62.1)	346 (59.0)	
More than five	239 (14.0)	77 (13.1)	
Living with parents			0.011*
No	188 (11.0)	88 (15.0)	
Yes	1516 (88.9)	499 (85.0)	
Cohesion among household members^2^			0.129
No	265 (15.5)	107 (18.2)	
Yes	1439 (84.5)	480 (81.8)	
Currently in a relationship			0.665
No	1189 (69.8)	404 (68.8)	
Yes	515 (30.2)	183 (31.2)	
IRSAD Quintiles^3^			0.695
Q1 (0-20%) - Most disadvantaged	421 (24.7)	135 (23.0)	
Q2 (20-40%)	347 (20.4)	118 (20.1)	
Q3 (40-60%)	395 (23.2)	153 (26.1)	
Q4 (60-80%)	246 (14.4)	84 (14.3)	
Q5 (80-100%) - Most advantaged	295 (17.3)	97 (16.5)	
Any medical condition^4^			0.036*
No	1322 (77.6)	435 (74.1)	
Yes	382 (22.4)	152 (25.9)	
Employment status during CRP^5^			0.047*
Unemployed	883 (51.8)	332 (56.6)	
Employed (part-time/full-time)	821 (48.2)	255 (43.4)	
Changed household composition^6^ during CRP			<0.001***
No	1464 (85.9)	466 (79.4)	
Yes	240 (14.1)	121 (20.6)	
Received coronavirus supplement^7^ during CRP			0.018*
No	1418 (83.2)	463 (78.9)	
Yes	286 (16.8)	124 (21.1)	

^1^ An individual aged 15 years and above are entitled to work (full-time/part-time) in Australia according to the Australian Labour Force

^2^ Cohesion - the ability of a family or household members to get along with each other

^3^ IRSAD - Index of Relative Socioeconomic Advantaged and Disadvantaged, is a general measure of both relative socioeconomic advantage and disadvantage at the area level. It uses a range of different Census variables including income, education, employment, occupation and housing characteristics.

^4^ Whether the youth have any conditions that lasted or are likely to last for 6-months or more (e.g., sight problems not corrected by glasses or contact lenses, difficulty learning or understanding things, limited use of limbs, any condition that restricts physical activity or disfigurement or deformity, and any mental illness for which help, or supervision is required).

^5^ CRP - Coronavirus Restriction Period, between March and May 2020.

^6^ Changed household composition - Whether the composition of household changed (any person added or moved out) during COVID-19 restriction except youth and their parents since the previous wave.

^7^ Coronavirus supplement - Additional income support during the COVID-19 pandemic from the Australian Government for people aged 15 and over, which included Youth Allowance for students and apprentices, and Youth Allowance for Jobseeker and/or JobKeeper

Pearson's chi-square test was used to explore whether there was a bivariate association between the predictor variables and outcome variable (COVID-19 testing)

Level of significance considered ***p<0.001, **p<0.01, *p<0.05

Results from logistic regression models of respondents being tested for SARS-CoV-2 testing are portrayed in Table 3. The strongest predictor of COVID-19 testing was living in major cities (aOR 1.64, 95% CI: 1.21–2.24) compared to those who were living in rural/remote areas. The older age group were 1.91 times (95% CI: 0.98–3.91) more likely to be tested for COVID-19 in comparison to the younger age group. Also, youth with any medical condition (aOR 1.31, 95% CI: 1.06–1.62) were more likely to get tested for COVID-19 compared to their counterparts.

**Table 3:** Logistic regression models for youth having been tested for COVID-19

Predictors of COVID-19 testing	Youth having been tested		VIF^#^
	Adjusted OR	95% CI	
Age (Ref. 16-17)			3.06
20-21	1.91*	0.98-3.91	
Remoteness (Ref. Rural/Remote)			1.03
Major cities	1.64**	1.21-2.24	
Education (Ref. Technical/Others)			2.61
Secondary	1.38	0.72-2.68	
Diploma and above	0.72	0.44-1.13	
Living with parents (Ref. Yes)			1.29
No	1.45	0.92-2.29	
Any medical condition (Ref. No)			1.01
Yes	1.31*	1.06-1.62	
Employment status during CRP (Ref. Unemployed)			1.07
Employed	0.84	0.67-1.04	
Changed household composition during CRP (Ref. No)			1.13
Yes	1.27	0.91-1.76	
Received coronavirus supplement during CRP (Ref. No)			1.25
Yes	1.08	0.81-1.46	
Model performance tests			
McKelvey & Zavoina's R^2^ statistic ^a^	0.72		
Goodness-of-fit test statistic ^b^ (p-value)	1.60 (0.208)		
Mean VIF (Max)	1.56 (3.06)		

Level of significance considered ***p<0.001, **p<0.01, *p<0.05

aOR=Adjusted odds ratio; CI=Confidence interval

^#^ VIF (Variance Inflation Factor) - an indicator of measuring multicollinearity; a VIF value more than 10 indicates a high correlation and VIF around 1 indicates no such correlation

^a^ McKelvey & Zavoina's R^2^ statistic - smaller than 1 indicates the model is well-fitted

^b^ Goodness-of-fit test statistic - p>0.05 indicates a good model fit

Further, Table 3 portrays results obtained from multiple diagnostic tests which ensure precise regression estimation. For example, McKelvey & Zavoina’s R^2^ statistic (less than 1.0) and Goodness-of-fit test statistics (*p* > 0.05) indicated the model was well-fitted. Also, the VIF mean of 1.56 indicated that there was no multicollinearity among the predictor variables used in the model.

## Discussion

This study provides population-based estimates of COVID-19 testing and examined the factors associated with COVID-19 testing in a nationally representative sample of Australian youths aged between 16 and 21 years using data from wave 9C1 from the LSAC.

Our study revealed that one-quarter of adolescents/young adults were tested in 2020 in the study sample. Studies demonstrate that the reasons youths do not participate in asymptomatic COVID-19 testing include concerns about the mental health impact of self-isolation, and the impact on others if the test is positive (such as forcing others into isolation), [34] and thinking that testing is a potential waste of resources [35]. Past research also suggests that barriers to testing may include misrecognition or misattribution of symptoms, logistical issues such as having no mode of transport or long wait times, discomfort surrounding nasopharyngeal sampling methods, concerns regarding the stigma of testing positive, costs associated with self-isolating, lack of trust in the government and health or socioeconomic vulnerability [36].

Our study found youth were more likely to get a COVID-19 test if they were living in major cities. This is because in Australia during the study period COVID-19 was predominantly in urban areas [37] given increased living density and connectivity to people [38]. In Australia, this is also because most cases (before June 2021) were imported from overseas and spread into the urban community from quarantine facilities based in cities. COVID-19 testing rates were low among people from rural areas compared to urban areas. This may be due to lower COVID-19 infection in rural Australia during the study period, and therefore rural people plausibly thought they didn’t need to test for COVID-19. Another hypothesis is that people living outside of urban areas may have less access to COVID-19 testing. Recent studies in the UK and Australia have found that one of the most common reasons for symptomatic individuals not getting tested was not knowing where to go to receive a COVID-19 test [39, 40]. COVID-19 testing rates for adolescents were lower in rural communities. Given the common combination of poverty, the higher prevalence of comorbid diseases, and poor accessibility to healthcare, [41] individuals in rural settings are likely to experience worse outcomes when COVID-19 outbreaks occur, as was the case for the Wilcannia outbreak in rural NSW, Australia [42].

Further, adolescents and young people with any pre-existing medical conditions were significantly more likely to have had a COVID-19 test. This is likely because evidence from the COVID-19 pandemic has demonstrated that individuals with pre-existing medical conditions are more likely to get severely ill with COVID-19 compared to those who do not have any prior condition [43–45]. Studies have also shown that people with disabilities are more prone to developing chronic diseases and are more vulnerable to COVID-19 infection, and if infected, ultimately face worse health outcomes [43, 46]. While 54% of those tested in our study sample were 16–17 years old, regression analyses demonstrated that increasing age is significantly associated with a higher likelihood of having had a COVID-19 test. This could be attributed to the fact that the risk of developing severe disease and dying from COVID-19 infection rises with increasing age [44, 47]. Though age might have less impact on young adults compared to aged individuals [48].

Employment during CRP was associated with higher COVID-19 test rates among Australian youth. We hypothesise this may be because if respondents were ill or were a close contact with someone with COVID-19 they may have been required to be tested for COVID-19 before returning to work [49]. Changes in family composition at the time of the CRP were also associated with higher test rates in youth. This may be an effort to protect household members [40, 50]. Government promotions have framed the motivation to be COVID-19 tested as helping the community rather than avoiding individual risk, [51] and have used identity-based messages such as “don’t be a spreader” [52]. Youth whose income was severely affected by COVID-19 and received an Australian Government financial supplement (JobSeeker, JobKeeper payment, Youth Allowances) during the CRP had higher testing rates. This may be because young people aged 15–24 years were seeking work, since the unemployment rate for this age group increased from 12 to 16% during the first COVID-19 lockdown between March and May 2020 [53]. However, it is essential to emphasize that the overall impact of COVID-19 on adolescents and young adults is complicated and not yet fully understood. As a result, more research is warranted to understand the cause of these associations [53].

Our study had the following limitations. First, the data were cross-sectional (i.e., only Wave 9C1 from the LSAC), therefore we were unable to evaluate the causation or temporality of the observed link between predictor variables and PCR COVID-19 testing in adolescents and young adults. Second, information regarding PCR COVID-19 testing was self-reported and so our results may be distorted by social-desirability bias. Third, the generalisability of findings may be limited to only young Australian people aged between 16 and 21 years . As well, the sample was not representative of the overall Australian population, and a degree of selection bias was present due to non-random participation. Fourth, we were also unable to gather data on whether respondents were COVID-19 close-contacts, if vulnerable people lived in the same household or were in regular contact, or if testing was a requirement of their employment. However, adolescents are the most likely group to have exposure to the virus as they are known to have a greater number of social contacts compared to other age groups, [53] and are less likely to be required to have COVID-19 testing than older age groups given lower levels of employment due to schooling. Moreover, the findings presented in our paper are factors related to PCR COVID-19 testing, regardless of exposure to the SARS-COV-2 virus or having COVID-19 symptoms at the time of testing. Factors related to other testing methods or testing for those known to be exposed to COVID-19 or with COVID-19 symptoms may differ. Another limitation may be the difficulty in comparing these findings to other countries. For example, Australia’s COVID-19 testing rate has consistently been higher than many other countries throughout the pandemic. During the study period, for instance, Australia was conducting COVID-19 tests at approximately 2.7 per day per 1000 people [54] compared to the US (1.9 per 1000), and the UK (1.3 per 1000) and India (0.654 per 1000) [55]. This may be due to Australia’s relatively high national wealth (and lower inequality compared to the US), as well as financial support for individuals and businesses afflicted by the pandemic [56]. When the data were collected for our study Australia had strict COVID-19 restrictions and its international border was closed. At the time, Australia had a low number of COVID-19 cases (i.e., a total of 28,408 COVID-19 cases in 2020 for Australia’s population of 26 million) [57] compared to other international countries (e.g., the UK had 2,48,8780 COVID-19 infections, population 67 million, [58] and Germany reported 1,71,9737 COVID-19 cases [59] in a populatio

n of 83 million in 2020). In 2020 the proportion of children and youth with COVID-19, compared to other age groups, was smaller than the proportion now in 2022.

## Conclusions

The current study indicates that three-quarters of adolescents and young adults did not get tested for COVID-19 in 2020 in Australia. For youths aged 16–21 years only, increased age, living in major cities and having a pre-existing medical condition were associated with higher testing rates. Employment, changed household composition and having received a governmental supplementary payment during the first lockdown in Australia were also found to be associated with increased COVID-19 testing in the same age-group.

## Supplementary Information

**Additional file 1.** Reporting checklist for cross sectional study.

## Data Availability

The LSAC data (10.26193/QR4L6Q) that support the findings of this study are available free of cost on request from the NCLD and ADA Dataverse, but restrictions apply to the availability of these data, which were used under license for the current study, and so are not publicly available. Instructions to apply for the LSAC datasets are available at https://growingupinaustralia.gov.au/data-and-documentation/accessing-lsac-data.

## Footnotes

1 Additional income support during COVID-19 pandemic from Australian Government, which included Youth Allowance students and apprentices, and Youth Allowance for JobSeeker or JobKeeper [36]. Service Australia. Coronavirus Supplement: Services Australia, Australian Government; 2021 [Available from: https://www.servicesaustralia.gov.au/individuals/services/centrelink/coronavirus-supplement.]
